# Can activated titanium interbody cages accelerate or enhance spinal fusion? a review of the literature and a design for clinical trials

**DOI:** 10.1007/s10856-021-06628-1

**Published:** 2021-12-18

**Authors:** Nathaniel Toop, Connor Gifford, Rouzbeh Motiei-Langroudi, Arghavan Farzadi, Daniel Boulter, Reza Forghani, H. Francis Farhadi

**Affiliations:** 1 Departments of Neurological Surgery, The Ohio State University Wexner Medical Center, Columbus, OH USA; 2 Department of Neurosurgery, College of Medicine, University of Kentucky, Lexington, KY USA; 3 Department of Radiology, The Ohio State University Wexner Medical Center, Columbus, OH USA; 4 Department of Radiology, McGill University, Montreal, QC Canada

## Abstract

While spinal interbody cage options have proliferated in the past decade, relatively little work has been done to explore the comparative potential of biomaterial technologies in promoting stable fusion. Innovations such as micro-etching and nano-architectural designs have shown purported benefits in in vitro studies, but lack clinical data describing their optimal implementation. Here, we critically assess the pre-clinical data supportive of various commercially available interbody cage biomaterial, topographical, and structural designs. We describe in detail the osteointegrative and osteoconductive benefits conferred by these modifications with a focus on polyetheretherketone (PEEK) and titanium (Ti) interbody implants. Further, we describe the rationale and design for two randomized controlled trials, which aim to address the paucity of clinical data available by comparing interbody fusion outcomes between either PEEK or activated Ti lumbar interbody cages. Utilizing dual-energy computed tomography (DECT), these studies will evaluate the relative implant-bone integration and fusion rates achieved by either micro-etched Ti or standard PEEK interbody devices. Taken together, greater understanding of the relative osseointegration profile at the implant–bone interface of cages with distinct topographies will be crucial in guiding the rational design of further studies and innovations.

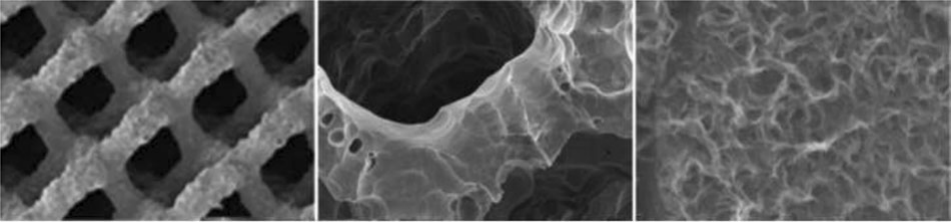

## Introduction

Solid bony fusion is an essential requirement for successful spinal arthrodesis surgery that allows for stability of adjacent vertebrae [1–4]. A complex balance of biological and mechanical factors is at play during interbody fusion following cage implantation [5]. Interbody cages are most commonly composed of either polyetheretherketone (PEEK) or titanium (Ti) [6]. While PEEK can cause a local inflammatory response that can interfere with osseous integration, solid titanium cages overcome this concern but alternatively can induce mismatch in the elastic modulus [7–11]. The use of both Ti and PEEK in clinical practice is limited by delayed and incomplete integration with adjacent bony surfaces following implantation. The recent development of novel porous cages has been guided by the goals of accelerating osseointegration of the cage to the adjacent vertebrae and thus reducing motion by enhancing the fusion of the cage at the interface with the bone [12, 13].

Enhancements to implant topography and architecture, including surface treatments and porous technologies, can putatively accelerate osseointegration and osteoconduction, which is key to long-term biomechanical function of interbody devices [11, 14, 15]. Further, interlaced lattice or scaffold designs can mimic cancellous bone structure through a microscopic porous implant–bone interface that can promote osteogenesis and bony fusion over the long-term [16–18]. Such complex lattice geometric designs and ability to scaffold biomaterial cannot be typically created by traditional manufacturing processes and are dependent on sophisticated 3DP technologies [17, 19, 20].

The purpose of this article is to critically assess the tissue engineering potential of modified implant surfaces in spinal surgery, in particular with respect to the ability of activated surface and scaffold designs to accelerate or enhance osseointegration and structural stability following spinal instrumented arthrodesis procedures. We review a new imaging modality, dual-energy computed tomography (DECT), which allows for additional image post-processing and material characterization not possible with conventional systems. Finally, we discuss the rationale for two recently initiated randomized clinical trials designed to evaluate the rate of early osseous fusion using DECT within either activated Ti or PEEK lumbar interbody cages. Taken together, we believe that new insights will soon be gained to help elucidate the potential relative efficacy of microscopic lattice structures in accelerating the process of osseointegration and fusion.

## Cage topography and enhanced osseointegration

While current lumbar interbody cage designs are generally associated with favorable radiologic and clinical outcomes [21–23], full bony integration is not always observed and is often delayed, in particular with concomitant smoking, older age, or immunosuppressant use [24–26]. Furthermore, given that mechanical loads regulate bone remodeling and maintenance, stress-shielding due to implant-bone stiffness mismatch can lead to eventual interface failure [27]. To overcome this limitation, novel technologies can take advantage of designing implants with more favorable mechanical and molecular properties, thus more closely resembling physiologic trabecular bone patterning.

A recent in vitro evaluation showed that implants bearing a trabecular scaffolded topography enhance cellular proliferation, bone matrix formation, and mineralization with increased mechanical strength by 20% for compression strength and 60% for compressive modulus [17]. Further, these implants exhibit expedited cellular proliferative activity as well as increased extracellular matrix production and ~50% higher calcium production as compared to standard templates [28, 29]. While these early studies are suggestive of potential efficacy, well-designed clinical studies will ultimately be required to confirm whether implant microstructure can serve as a more effective medium to support healing and osseous fusion [30].

One critical implant property that can play a role in osteoconduction and osseointegration is the three-dimensional geometry and intricately designed porosity. Specifically, interbody cage porosity accommodates bone ingrowth with the potential to yield improved implant fixation [27, 31]. A minimum pore size of 100 μm is more conducive to bony ingrowth than sizes less than 75 μm, while pore sizes over 300 μm further enhance the osteogenic response [32, 33]. In a transcortical rabbit study, increasing pore sizes from 100 to 300 μm not only increased bony integration within the pores of titanium alloy implants but also enhanced the percentage of lamellar bone that was produced [18]. Another study in sheep compared a porous titanium cage (with median 68% porosity and 710 μm pore size) to the standard PEEK cage. The porous titanium cage promoted enhanced osseointegration and achieved more rapid bone growth [34]. In yet another ovine study, 3DP porous titanium cages demonstrated increases in bone ingrowth as compared with solid PEEK and plasma-sprayed porous Ti-coated PEEK [16]. The beneficial impact of adding porosity to the implant is not limited to Ti, as porous PEEK cages have also been shown to enhance osseointegration [35, 36]. In vitro evaluation has confirmed favorable attachment, proliferation, and mineralization of cells cultured on porous PEEK versus either smooth PEEK or smooth Ti surfaces [36]. At the implant level, in vivo studies have identified comparable bone ingrowth into porous PEEK as that previously reported for porous Ti, leading to twice the fixation strength as compared to smooth PEEK implants [36].

Implant surface roughness and surface finish also may play a significant role in promoting osseointegration [37]. An early study showed that a roughened surface having Ra measurements ranging from 5.0 to 11.8 was attained via a surface-blasting process with the effect of increasing overall bone-implant contact, on the surface and within the pores [18]. Further work has revealed that ‘microroughness’ influences the types of integrins that are produced by the cells at the interface, promoting those subunits associated with bone proteins, such as α2 and β1, but not those subunits associated with soft tissue proteins, such as α5 or αv. Thus, microroughness can affect the progression of the osteoblast phenotype by up‐regulating specific integrins that directly regulate osteoblast differentiation and local factor production [38]. Most recently, nanostructured surfaces, i.e. those containing irregularities less than 100 nm, have been posited to more faithfully mimic the nanoarchitecture of natural tissues [39, 40].

Another study compared osseointegration when alumina and a multiphase calcium phosphate (MCD) were used as the blast media. Blasted Ti alloy (Ti6Al4V) cylinders were implanted into rabbit tibiae and then evaluated two and four weeks after implantation. The histologic results showed the MCD to elicit significantly greater volume of new bone formation [41]. A more recent study demonstrated that increased surface roughness enhances in vitro osteoblast maturation and production of local factors associated with osteogenesis and in vivo that the same topographies increase bone‐to‐implant contact and torque removal forces [42].

Intriguingly, the combination of nanostructures onto microroughened surfaces may synergistically enhance the production of osteoblast differentiation markers and local factors important for bone formation compared to unmodified microrough controls [43]. Indeed, osteoblasts have been shown to exhibit a more differentiated phenotype when cultured on Ti6Al4V surfaces with a microtopography that includes submicron and nanoscale features [44].

Yet another factor that can influence the biomimetic properties of the bone–implant interface is the modulus of elasticity. Specifically, 3DP can design implants with an elasticity modulus resembling natural or cadaveric tissue. As a result, a better elastic recovery, biodegradability and cytocompatibility is expected [45, 46]. Moreover, implants with modified elasticity modulus also show enhanced mechanical characteristics in compression and shear when compared with standard designs, resulting in better device–tissue interface and biocompatibility [47].

A number of activated Ti cages are commercially available but with no long-term clinical evidence supporting their relative efficacy in promoting early osseointegration and fusion [48, 49]. A recent prospective study evaluated novel 3DP interbody devices in 40 patients (total of 53 segments) undergoing posterolateral interbody fusion (PLIF) procedures. Bone fusion results were promising with 94.3%, 86.7%, and 94.3% of interbody segments showing complete anterior, posterior, and bilateral bone bridges on sagittal and coronal computed tomography (CT) views, respectively. No pseudarthrosis was noted and no revision surgeries were required [50]. Finally, while the potential benefits of accelerated osseointegration and bony fusion are particularly relevant for patients with anticipated compromised bone fusion (such as elderly patients with osteoporosis, or with concomitant tobacco or immunosuppressant use), no controlled studies have yet been undertaken.

## Rationale and design of RCTs

Clinical confirmation of enhanced osseointegration or bony fusion through activated Ti cages remains lacking. As such, the principal author (HFF) has designed two separate randomized double-arm single-blinded studies in patients undergoing either single- or double-level instrumented arthrodesis procedures (Trial 1: NCT0364751, Trial 2: clinical trials.gov submission in progress) to compare radiologic and clinical outcomes using either activated Ti cages (Trial 1: banana-shaped Nexxt Spine Matrix^®^ or Trial 2: straight-shaped Medtronic Adaptix^TM^) versus PEEK cages.

**Fig. 1:**
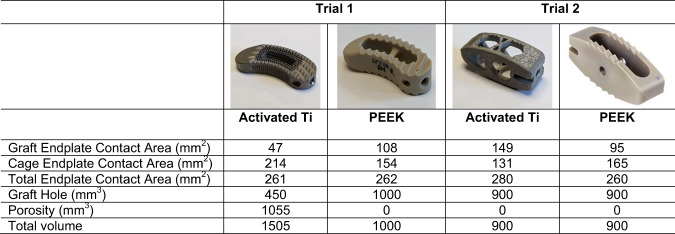
Pictures of Activated Ti and PEEK interbody cages used in Trial 1 and 2 (left and right) along with their respective surface contact characteristics. For Trial 1, considering 34 mm length × 10 mm width (6° lordotic) Ti and PEEK implants, comparable graft and cage endplate contact areas (ECAs) are noted. Similarly for Trial 2, considering 26–28 mm length × 10 mm width Ti and PEEK implants, comparable graft and cage ECAs are also noted

**Fig. 2:**
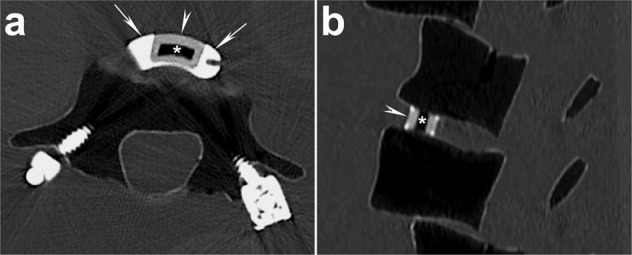
Appearance of titanium cage on dual-energy CT (DECT) imaged in a synthetic polyurethane foam/ hydroxyapatite lumbar spine model. **a** Axial and (**b**) sagittal reformatted 100 keV virtual monochromatic images (VMIs) reconstructed from a DECT scan of the spine show that the lattice portion has high density (arrowheads) but that is less than the solid titanium portions (marked by the arrows). The center is hollow (asterisks). Scan acquired using a rapid kV-switching CT scanner (Revolution CT, GE Healthcare)

Both Ti cages have hollow centers and are characterized by similar cage, graft, and total endplate contact areas to the respective PEEK cages (see Fig. 1). Both cages also incorporate proprietary macro- and nano-scale biomimetic surface texturing that both promote initial fixation and bone remodeling required for osseointegration. The architecture of the cage in Trial 1 further incorporates a lattice that provides a consistent 70% porosity and an interconnected array of 300–700μm pores with two specific geometries; the inferior/superior surfaces incorporate a square mesh between the exterior structural frame while the lateral aspects employ a coupled hexagon-diamond mesh. Both lattice geometries were designed so that the resulting pore sizes would range from 300–700 μm, which is a dimensional geometry purported to be conducive to osseointegration. The appearance of the Trial 1 Ti interbody cage on CT is shown in Fig. 2.

Starting in August 2018, enrolled subjects have been randomized to receive either an activated Ti or PEEK cage (Trial 1) implanted at each level of single- or contiguous double-level lumbar arthrodesis procedures. Both cages will be used in conjunction with milled local autograft bone generated as part of the spinal decompression portion of the procedure. Clinical and radiographic outcomes are being collected at regular intervals post-operatively. The primary outcome measure of effectiveness is radiographic bony fusion as assessed by an independent neuroradiologist at 6 months post-operatively, which grossly corresponds to the early phase of expected fusion following lumbar interbody cage placement. Radiographic bone fusion will be graded by the method of Brantigan and Steffee as modified to describe the Fraser definition of locked pseudoarthrosis (BSF scale) with a Grade BSF-3 being considered successful bone fusion. Grade BSF-3 is defined by radiographical fusion presenting bone bridges within at least half of the fusion area with at least the density originally achieved at surgery (see Table 1) [51–53].

**Table 1:** Radiographic assessment of interbody fusion by the method of Brantigan, Steffee, and Fraser (BSF)[53]

BSF-1: Radiographical pseudarthrosis is indicated by collapse of the construct, loss of disc height, vertebral slip, broken screws, displacement of the carbon cage, or significant resorption of the bone graft, or lucency visible around the periphery of the graft or cage.
BSF-2: Radiographical locked pseudarthrosis is indicated by lucency visible in the middle of the cages with solid bone growing into the cage from each vertebral endplate.
BSF-3: Radiographical fusion: bone bridges at least half of the fusion area with at least the density originally achieved at surgery. Radiographical fusion through one cage (half of the fusion area) is considered to be mechanically solid fusion even if there is lucency on the opposite side.

## Radiologic evaluation with dual-energy CT

**Fig. 3:**
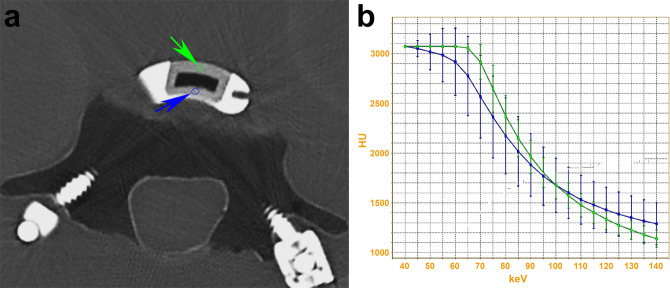
Example of energy-dependent density (attenuation) changes of the titanium cage lattice using dual-energy CT (DECT). **a** Axial image demonstrates two regions of interest (ROIs) with the corresponding (**b**) spectral Hounsfield Unit attenuation curves (SHUACs). ROIs are used to obtain quantitative information from the image. The small circles represent the ROIs (to which the arrows point) and correspond to the regions where the measurements were obtained. The information obtained from the ROIs is illustrated in the corresponding SHUACs, which demonstrate the changes in material density at various virtual monochromatic image (VMI) energies. By way of comparison with conventional single energy CT acquisition, there would only be a single attenuation per ROI, typically corresponding to that obtained on the 65–70 keV VMIs. Thus, DECT allows the energy-dependent characteristics of various materials to be exploited to improve diagnostic evaluation. Scan acquired using a rapid kV-switching CT scanner (Revolution CT, GE Healthcare)

Patients enrolled in these trials will be assessed with an advanced form of CT, called DECT [54–59]. While a detailed discussion is beyond the scope of this article, a brief review will be provided to illustrate the potential of DECT to achieve new insights in imaging of spinal fusion with cages. DECT confers the same advantages as conventional, single-energy CT (SECT) such as rapid scan acquisition times, a high spatial resolution, and the ability to demonstrate and evaluate fine bone detail. However, DECT also enables additional image post-processing and material characterization not possible with conventional CT systems. With SECT, a polychromatic beam at one peak energy is emitted by the X-ray tube, passes through the patient, and the attenuation data are captured by a detector array. With DECT, attenuation data are instead obtained separately at both a low and a high energy. In this fashion, the data are combined and can be processed to generate images or evaluate tissue characteristics in unique ways, taking advantage of energy-dependent attenuation (i.e. density or brightness on CT) of different tissues (Fig. 3).

**Fig. 4:**
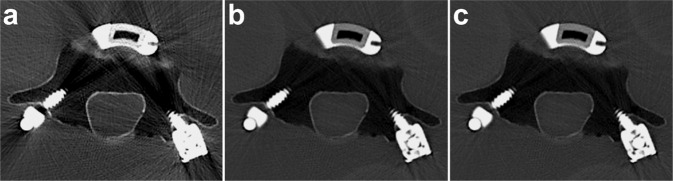
High energy virtual monochromatic image (VMI) reconstructions allow for artifact reduction. Axial (**a**) 70 keV, (**b**) 100 keV, and (**c**) 140 keV VMIs reconstructed from the same dual-energy CT (DECT) scan are shown. The images are displayed using the same window-level display settings to enable a direct and unbiased comparison. Note the significant reduction in artifact and image noise on the high energy VMIs at 100 keV (**b**) or 140 keV (**c**) compared to the 70 keV VMI (**a**), which is typically considered to correspond to the conventional single energy CT scan. Of note, the images shown here are the qualitative correlates of the attenuation measurements shown in Fig. 3

Unlike with current X-ray tube technology where greater artifact is expected with a polychromatic beam, DECT can generate virtual monochromatic images (VMIs) (Figs. 2–4) [54–59]. Using sophisticated algorithms, the scanned data at different energies are combined to generate images at a range of prescribed X-ray energy levels and thus simulate what would be expected with a monochromatic X-ray beam. VMI energies that can be generated with current DECT systems typically range between 40 and 140 keV (Fig. 3), with VMIs of 65–70 keV generally considered similar to 120 keV on standard acquisition [60–62].

With current X-ray tube technology, it is not possible to generate monochromatic spectra for scanning and greater artifact is expected with a polychromatic beam. As such, the potential for diminished artifact stems from the ability of VMIs to simulate what would be expected with a monochromatic X-ray beam. Furthermore, VMI images can be generated at a wide range of energies with unique advantages depending on the problem at hand and material of interest (Fig. 3). By generating VMIs at energies higher than those typically used to simulate a standard acquisition, one can reduce artifact arising from metallic hardware [63–67] (see Fig. 4). This is clearly of interest in imaging of the instrumented spine and the primary motivation for using DECT for patient follow-up in these two trials.

Finally, DECT scans can be used to generate basis material decomposition maps. While the feasibility depends on the specific material of interest and its physical properties, these maps can be used to estimate the distribution and concentration of certain materials, which is not possible using conventional CT imaging. One such example, virtual non-calcium maps, are used to demonstrate marrow edema [64, 68, 69]. In contrast to conventional SECT, multiple studies have demonstrated that DECT can demonstrate marrow edema with high accuracy, improving detection of subtle findings such as non-displaced fractures [68]. Thus, in addition to the use of different energy VMIs, one can exploit DECT to improve visualization of bony fusion beyond artifact reduction alone. For example, one can leverage the material specific energy-dependent properties and create DECT maps that are tailored for tracking and demonstrating osseointegration within a cage lattice using basis material decomposition. Since all of the above are post-processing maneuvers based on the source data acquired at the time of DECT scanning and do not require any additional patient scanning or radiation exposure, they represent exciting additional applications that will be investigated during the course of these trials.

## Conclusions

Enhanced implant-bone integration and fusion rates may have significant clinical benefits, particularly in more complicated clinical scenarios where fusion may be delayed, such as in osteoporosis, smokers, or with concomitant immunosuppressant use. The results of these two trials should provide relevant information regarding the relative efficacy of activated Ti surfaces (without or with a supplemental lattice network) in accelerating the process of osseointegration and fusion. Novel insights in this regard are likely to be gained with DECT imaging, whose potential advantages include enhanced artifact reduction. Our conclusions should also assist in the design of further multicenter studies evaluating potential clinical benefits, either for similar degenerative conditions or for more complex reconstructions such as in adult spinal deformity.
